# Predictive models of post-traumatic stress disorder, complex post-traumatic stress disorder, depression, and anxiety in children and adolescents following a single-event trauma

**DOI:** 10.1017/S0033291724001648

**Published:** 2024-09

**Authors:** Jessica Memarzia, Katie Lofthouse, Tim Dalgleish, Adrian Boyle, Anna McKinnon, Clare Dixon, Patrick Smith, Richard Meiser-Stedman

**Affiliations:** 1 Department of Clinical Psychology & Psychological Therapies, Norwich Medical School, University of East Anglia, NR4 7TJ, Norwich, UK; 2 Medical Research Council Cognition and Brain Sciences Unit, University of Cambridge, Cambridge, UK; 3 Cambridgeshire and Peterborough NHS Foundation Trust, Cambridge, UK; 4 Emergency Department, Addenbrooke's Hospital, Cambridge, UK; 5 Centre for Emotional Health, Department of Psychology, Macquarie University, Sydney, NSW, Australia; 6 Sussex Partnership National Health Service Foundation Trust, Sussex, UK; 7 Department of Psychology, Institute of Psychiatry, Psychology and Neuroscience, King's College London, London, UK

**Keywords:** adolescents, anxiety, children, depression, predictors, PTSD, trauma

## Abstract

**Background:**

This study examined the power of theory-derived models to account for the development of PTSD, Complex PTSD (CPTSD), depression, and anxiety in children and adolescents who had experienced a single-event trauma.

**Methods:**

Children (*n* = 234, aged 8–17 years) recruited from local Emergency Departments were assessed at two and nine weeks post-trauma. Data obtained from self-report questionnaires completed by the child, telephone interviews with parents, and hospital data were used to develop four predictive models of risk factors for PTSD, CPTSD, depression, and Generalized Anxiety Disorder (GAD). ICD-11 proposed diagnostic criteria were used to generate measures for CPTSD and PTSD to assess for risk factors and identify the sample prevalence of these disorders.

**Results:**

At nine weeks post-trauma, 64% did not meet criteria for any disorder, 23.5% met criteria for PTSD, and 5.2% met criteria for CPTSD. 23.9% and 10.7% had developed clinically significant symptoms of depression and GAD, respectively. A cognitive model was the most powerful predictive model, a psychosocial model was weak, and subjective markers of event severity were more powerful than objective measures.

**Conclusions:**

Youth exposed to single-incident trauma may develop different forms of psychopathology, and PTSD and CPTSD are frequently experienced alongside other conditions. The cognitive model of PTSD shows utility in identifying predictors of PTSD, CPTSD, depression, and GAD, particularly the role of trauma-related negative appraisals. This supports the application of cognitive interventions which focus upon re-appraising trauma-related beliefs in youth.

## Introduction

Numerous psychopathological outcomes have been studied following trauma in children, including PTSD or acute stress disorder (ASD), depression, conduct and behavioral difficulties, separation anxiety, phobias, and generalized anxiety disorder (GAD). Research typically focuses on PTSD, with other psychopathology studied as comorbidities or secondary outcomes of PTSD (Goenjian et al., 1995). However, research in adults indicates that individuals may develop other disorders, such as phobias or depression, and not just PTSD symptoms (Ehring, Ehlers, & Glucksman, 2008; O'Donnell, Creamer, & Pattison, 2004).

In response to concerns that PTSD doesn't encapsulate the full extent of reactions to more repeated or severe forms of trauma exposure, the 11th edition of the International Classification of Diseases (ICD-11) included ‘complex PTSD’ (CPTSD) as a new diagnosis. To meet criteria for a CPTSD diagnosis, in addition to the core PTSD criteria (re-experiencing, avoidance, and perceived threat), three CPTSD-specific symptom clusters known as disturbances in self-organization (DSO) must be met: affect dysregulation, negative self-concept, and interpersonal difficulties. Childhood interpersonal trauma is a risk factor for developing CPTSD compared to PTSD, with a dose-response type relationship where exposure to multiple forms of interpersonal trauma increases risk of CPTSD (Hyland et al., 2017a). However, it has been argued that CPTSD can develop in response to a single traumatic stressor (Maercker et al., 2013); multiple traumas may therefore be best conceptualized as a risk factor, rather than a requirement, for CPTSD (Hyland et al., 2017b; Sachser, Keller, & Goldbeck, 2017).

Most research has focused on confirming CPTSD as a valid diagnosis and distinct from PTSD in adults (Cloitre, Garvert, Brewin, Bryant, & Maercker, 2013; Cloitre, Garvert, Weiss, Carlson, & Bryant, 2014; Elklit, Hyland, & Shevlin, 2014; Knefel & Lueger-Schuster, 2013; Knefel, Garvert, Cloitre, & Lueger-Schuster, 2015; Perkonigg et al., 2016). Two studies have validated CPTSD in children and adolescents (Perkonigg et al., 2016; Sachser et al., 2017).

Ehring et al. (2008) highlighted the need to investigate predictors differentiating between the development of various psychopathological presentations following trauma. They demonstrated the utility of cognitive theories of emotional disorders in differentiating between PTSD, travel phobia, and depression in adults following motor-vehicle accidents. To date, no study has utilized similar methodology to assess predictive models in understanding the risk factors of youth developing psychopathology following trauma.

### Models and predictors of PTSD and CPTSD

The evidence available for psychosocial predictors of PTSD in children is variable, with meta-analyses indicating the need for further assessment. Social support, prior life events, low intelligence, socioeconomic status, low self-esteem, and female gender were shown as consistent predictors of PTSD, but with only small to medium effect sizes, and younger age was found not to be a predictor of PTSD in youth (Cox, Kenardy, & Hendrikz, 2008; Trickey, Siddaway, Meiser-Stedman, Serpell, & Field, 2012).

Various psychosocial risk factors are considered in relation to adult CPTSD, including multiple prior traumas, interpersonal traumas, and interpersonal stressful life events (Herman, 1995; Hyland et al., 2017a), but research assessing these as CPTSD predictors is limited. Exposure to child abuse and multiple types of abuse increases an adult's likelihood of CPTSD *v.* PTSD (Cloitre et al., 2013; Powers et al., 2017). In youth, female gender and interpersonal trauma predict CPTSD *v.* PTSD (Sachser et al., 2017).

Ehlers and Clark (2000), Foa, Steketee, and Rothbaum (1989), and Brewin, Dalgleish, and Joseph (1996) proposed cognitive models of PTSD in which trauma memories and associated cognitive processes are key. Poor social support, prior or ongoing trauma, aversive secondary emotions, trauma severity, and prior psychopathology are risk factors to inhibition of adaptive processing of trauma memories, and thus PTSD.

The predictive power of the cognitive model has been demonstrated in children experiencing road traffic accidents (RTAs); 53–65% of the variance in PTSD symptoms was predicted by models including data-driven processing, negative appraisals of the trauma, rumination, and thought suppression (Ehlers, Mayou, & Bryant, 2003; Meiser-Stedman, Dalgleish, Glucksman, Yule, & Smith, 2009a; Stallard & Smith, 2007). Cognitive models are also thought to be disorder specific in predicting the psychopathology outcomes of experiencing a trauma; despite overlaps in symptoms and risk factors across PTSD, depression, and other anxiety disorders, cognitive factors differentiate whether an individual may develop one disorder presentation over another (Ehring et al., 2008). For example, poor trauma memory quality is likely to be mostly strongly associated with PTSD over depression or anxiety, and safety-seeking behaviors are thought to be present for PTSD and anxiety but not for depression. Other key cognitive mechanisms such appraisals and rumination may be strongly related to both PTSD and depression. However, data from adult studies is supportive of a role for disorder-specific content for each process: PTSD is more associated with trauma-specific appraisals (perceiving intrusive memories as a sign of permanent psychological damage) and rumination (asking why the trauma happened, what might have been done differently), while depression is more associated with mood-specific appraisals (such as perceptions of worthlessness) and rumination (a persistent focus on the reasons why someone might have low mood) (Beierl, Böllinghaus, Clark, Glucksman, & Ehlers, 2020; Ehring et al., 2008; Kleim, Ehlers, & Glucksman, 2012). The identification of such processes has implications for treatment, e.g. the importance of memory processing (through techniques such as imaginal reliving, *in vivo* or elaborating a trauma narrative) for the successful treatment for PTSD and the need to address mood-related rumination for the treatment of depression.

Both objective and subjective appraisals of event severity have been researched, with poor differentiation and little consistency in what constitutes markers of severity (Trickey et al., 2012). Recent research demonstrates that markers of event severity may include: interpersonal (*v.* non-interpersonal) trauma; the event resulting in a death; injury severity; levels of pain; and peritraumatic dissociation, perceived threat, fear, and panic responses (Cox et al., 2008; Ozer, Best, Lipsey, & Weiss, 2003; Saxe et al., 2005; Trickey et al., 2012; Vogt, King, & King, 2007).

### Aims of the current study

There have been few studies comparing predictive models across psychopathological outcomes of trauma, and none focused on youth. This, alongside the addition of CPTSD as a diagnostic category, warrants exploration of predictors of psychopathological outcomes of trauma-exposed youth.

The present study used data collected from a prospective longitudinal study of PTSD in children and adolescents following a recent trauma. Data pertaining to the course of DSM-IV, DSM-5, ICD-10, and ICD-11 PTSD and CPTSD has been reported (Elliott et al., 2021; Meiser-Stedman et al., 2017), as has data regarding the trajectory of PTSD symptoms (Meiser-Stedman et al., 2019). The present study aimed to identify risk factors for PTSD, CPTSD, depression, and anxiety at follow up. Specifically, the goodness of fit of predictive models of PTSD and CPTSD in youth, in comparison to depression and anxiety, were assessed, and predictors of these disorders explored. Predictive models were developed based on psychosocial factors, cognitive factors, subjective event severity, and objective event severity factors.

### Hypotheses

Firstly, it was hypothesized that peri- and post-trauma factors would be greater predictors of PTSD and CPTSD than pre-trauma psychosocial factors. Secondly, it was hypothesized that the cognitive model would have the best model fit in predicting PTSD and CPTSD. Finally, it was hypothesized that the cognitive model would have more power than other models in differentiating between PTSD, CPTSD, depression, and anxiety as trauma outcomes in youth.

## Methods

### Participants

Two hundred and sixty 8–17-year-olds were consecutively recruited from four Emergency Departments (ED) in the East of England between September 2010 and April 2013, who were identified by research nurses as presenting due to a single event trauma (e.g. a motor vehicle collision or assault), defined in accordance with DSM-V criteria (American Psychiatric Association, 2013), i.e. ‘exposure to actual or threatened death, serious injury, or sexual violence’.

Staff identified 774 eligible children; 168 (21.7%) could not be contacted; of 605 families contacted, 315 (52%) did not wish to participate, 30 (5%) did not meet eligibility criteria, and 260 (43%) agreed to participate. Initial assessments at two weeks post-trauma (T1) were completed by 226 participants, and 234 completed the assessment at nine weeks post-trauma (T2), with 260 participating in at least one timepoint. There were no significant differences between responders and non-responders on age, gender, ethnicity, or measures of injury severity and hospital treatment. However, responders were more likely than non-responders to experience more pain, admission to hospital, and to have experienced an assault (*v.* other) trauma (Meiser-Stedman et al., 2017).

Inclusion criteria were: exposure to a single discrete traumatic stressor defined in accordance with DSM-V criteria. Exclusion criteria were: intellectual disability; non-fluency in English; unconsciousness longer than 15 min following the event; a history of brain damage or moderate to severe traumatic brain injury as a result of the trauma; assaults involving a caregiver/close relative as the assailant; ongoing exposure to threat; any significant risk of self-harm or A&E attendance resulting from deliberate self-harm; being under the care of social services or a child protection issue related to the presentation; any current symptoms of PTSD following a previous trauma; unable to gain consent from parent/guardian. No participants were excluded based on their presentation being due to ongoing trauma. See online Supplementary Table S1 for data pertaining to the number of young people excluded based on each exclusion criterion.

### Measures

Self-report questionnaires and structured interviews were completed by parents and children two weeks post-trauma and hospital data was gathered by nurses in the ED at point of admission. See online Supplementary Table S2 for a summary of measures.

#### Psychosocial factors

Participant demographic data were collated with admission information from the hospital, including trauma type and injury characteristics. Parents' education level was categorized as those achieving up to GCSE or equivalent and those achieving higher education or training. Parents were asked about prior traumas experienced over their child's lifetime and life stressors over the past year using a list of possible events (see online Supplementary Table S3) taken from the Posttraumatic Diagnostic Scale (PDS-5; Foa, Cashman, Jaycox, & Perry, 1997; Foa et al., 2016); total number of prior traumas and life stressors were used as putative predictor variables. Prior poor well-being was identified by parents answering positively to: ‘Before the trauma, have you had concerns for your child's emotional well-being (e.g. anxiety, depression, or emotional problems)?’. Children's perception of their social support and quality of their relationships was assessed using a self-report questionnaire, the Multidimensional Scale of Perceived Social Support (MSPSS: Zimet, Dahlem, Zimet, & Farley, 1988; Cronbach's *α* = 0.93).

#### Cognitive factors

The cognitive model included cognitive processing during the trauma (Children's Data-Driven Processing Questionnaire [CDDPQ]: McKinnon, Nixon, & Brewer, 2008; Cronbach's *α* = 0.89); negative trauma-related appraisals (Child Post-traumatic Cognitions Inventory [CPTCI]: Meiser-Stedman et al., 2009a; Cronbach's *α* = 0.95); trauma memory quality (Trauma Memory Quality Questionnaire [TMQQ]: Meiser-Stedman, Smith, Yule, & Dalgleish, 2007; Cronbach's *α* = 0.83); post-traumatic dissociation (four-item questonnaire; Cronbach's *α* = 0.78); trauma-related rumination (three-item scale, Cronbach's *α* = 0.77); and self-blame (two-item scale, with Cronbach's *α* = 0.91; Meiser-Stedman et al., 2017).

#### Subjective event severity

This model focused on peritraumatic processes, including: panic responses (Child Peritraumatic Panic scale (CPP): Meiser-Stedman et al., 2017; Cronbach's *α* = 0.72); peritraumatic dissociation (four-item questionnaire; Cronbach's *α* = 0.67); and three items entered individually assessing peritraumatic perceived threat and fear (Meiser-Stedman et al., 2009a, 2009b; Cronbach's *α* = 0.76).

#### Objective event severity

This model used information gathered from the child's presentation at the ED including the number of injuries sustained, whether they had sustained a head injury, whether they were given opiate pain-relief in ED, and whether they were admitted to hospital. The child's rating of pain during the event was included (‘How much pain were you in at the time of the accident?’ with Likert responses on a four-point scale).

#### Assessment of outcomes

Outcomes were assessed at nine weeks post-trauma. PTSD symptoms were assessed using items from the Child PTSD Symptoms Scale (CPSS; Foa, Johnson, Feeny, & Treadwell, 2001). CPTSD DSO (disturbances of self-organization) symptoms were assessed using items drawn from the CPSS, the CPTCI (Meiser-Stedman et al., 2009b) and the self-blame questionnaire items (Meiser-Stedman et al., 2017), with PTSD and CPTSD diagnoses following the ICD-11 criteria derived from these; a symptom was present if the corresponding item scored one or higher (once per week or more, Sachser et al., 2017). Continuous measures for these outcomes were also derived: a nine-item PTSD measure from the CPSS (possible score range 0–27) and an eight-item CPTSD-DSO measure using items from the CPSS, CPTCI, and self-blame items (possible score range 0–24), excluding core PTSD symptoms to prevent multicollinearity. The internal consistency was Cronbach's *α* = 0.90 and 0.78 for the PTSD and CPTSD-DSO scales, respectively. The PTSD scale score showed good correlation (Pearson's correlation coefficient = 0.61) with a diagnostic measure of DSM-IV PTSD, assessed by the Children's PTSD Inventory (Saigh et al., 2000) semi-structured interview. The correlation between the PTSD scale and the CPTSD-DSO scale was 0.63. Full diagnostic criteria were used to identify frequencies of likely PTSD and CPTSD diagnoses at week nine. See online Supplementary Table S4 for item list and construct derivation.

The Short Mood and Feelings Questionnaire (SMFQ, Angold, Costello, Messer, & Pickles, 1995; Cronbach's *α* = 0.92) was used to assess depression symptoms, with a score of 8 or above indicating ‘likely depression’. The Spence Children's Anxiety Scale (SCAS; Spence, 1998) Generalised Anxiety Disorder (GAD) subscale (Cronbach's *α* = 0.87) was used to assess GAD symptoms, with computed *t* scores above 60 indicating ‘likely GAD’. These are well-validated measures in children and adolescents (Sharp, Goodyer, & Croudace, 2006; Spence, Barrett, & Turner, 2003).

### Study procedure

The study was approved by the UK National Research Ethics Service, Cambridgeshire 1 Research Ethics Committee (10/H0304/11). Participants were recruited after presenting at an ED; parents/caregivers of children who met eligibility criteria were contacted by letter enclosing information sheets and contacted by telephone one week after ED attendance. Informed consent from the parent and assent from the child was gained if eligibility criteria were met. Approximately two weeks following their trauma (T1), participants and parents were interviewed via telephone and asked to complete self-report questionnaires. Participants were assessed a second time approximately nine weeks post-trauma (T2), with the same interview and self-report measures.

### Analyses

Data processing and analysis was completed in Stata/IC Version 13.1 (StataCorp, 2013) and R 4.3.1, including the use of the simpleboot package (Peng, 2019). Missing data codes were assigned to ensure correct treatment of missing data by Stata. Stata uses complete case analysis by default (observations with any missing data are excluded), which is a valid method of treating missing data if it is deemed ‘missing at random’ (MAR) and if data missingness is independent of the outcome of interest. Due to different numbers of participants at each timepoint, many observations were ‘missing’ in the predictor or outcome variables. Data available from ED admissions were also variable. *t* tests were run to confirm no significant differences in outcome measures between participants with missing and complete data and there were no significant differences between complete cases and non-complete cases across both time points. Therefore, complete case analysis was deemed valid. Some differences in the model goodness of fit statistics may have partially reflected the different number of observations included in the analysis.

Pre-analysis screening of the normality, skew, and homoscedasticity of the data was completed. Many variables were skewed, residuals were not normally distributed, and the variance of residuals was heteroscedastic, violating parametric test assumptions, therefore non-parametric or other appropriate considerations were made.

Spearman's rank correlation coefficients (rho) were used to ascertain association strength between all continuous variables, and point biserial correlation coefficients were computed for dichotomous variables to identify correlations between predictor variables and outcome variables and identify any multicollinearity between predictor variables. For predictive model analysis, non-parametric adjustments were made to multiple linear regression models using bootstrapping, allowing for estimation of coefficients and standard errors (Chernick, 2008). To generate standardized coefficients, variables were transformed into standardized formation, and the regression was re-run to generate a beta coefficient equivalent (Acock, 2008). Both unstandardized and standardized coefficients are reported.

The predictive power and goodness of fit of the predictor models were compared by computing Akaike and Bayesian Information Criteria (AIC and BIC) and adjusted R-squared values. Low AIC and BIC values indicate better model fit, and higher *R*^2^ values indicate a greater proportion of variance accounted for by the predictor variables (Akaike, 1998; Raftery, 1995).

#### Statistical power

*N* > / = 50 + 8 m (where *m* = number of predictors) was used to calculate the required sample size for a reasonably powered multiple regression analysis (Green, 1991). For seven predictor variables, 106 participants would be required. To detect a large effect size with seven predictor variables and a power of 0.8 (alpha = 0.05) using a parametric multiple correlation analysis, 44 participants are required, and to detect a medium effect size, 103 participants are required (Green, 1991). A minimum of 189 participants were included in the analyses and no more than seven predictor variables were used in each model.

## Results

### Sample characteristics

Of the 260 participants, 118 (45%) had experienced a road traffic collision, 43 (17%) an assault, 82 (32%) an accidental injury, 15 (6%) a dog attack, and 2 (1%) had an acute medical emergency. Sample characteristics including age (mean 13.9 years), gender (43.5% female), psychosocial features, trauma characteristics, and predictor and outcome variables are summarized in Table 1.

**Table 1.** Correlations between week two predictor variables and outcomes at week nine post trauma

			Week nine outcomes			
	Mean (s.d.)/ frequency (%)	Range	PTSD	CPTSD	Depression	GAD
Outcomes at week nine						
PTSD	4.6 (5.9)	0–24	1			
CPTSD	2.9 (3.9)	0–21	0.54***	1		
Depression	4.5 (5.4)	0–23	0.56***	0.69***	1	
GAD	45.9 (9.1)	40–100	0.54***	0.58***	0.67***	1
Psychosocial factors						
Age	13.9 (2.9)	8.0–17.9	−0.09	0.08	0.02	0.13*
Female gender	108 (42.5%)	n/a	0.08	0.02	0.11	0.14*
Mother's education	147 (58.3%)	n/a	0.02	0.05	0.07	0.10
Freq. of prior traumas	0.9 (1.0)	0–5	0.06	0.14*	0.15*	0.15*
Freq. of prior life stressors	0.9 (1.2)	0–6	0.14*	0.10	0.09	0.20**
Prior wellbeing concerns	62 (24.2%)	n/a	0.08	0.19**	0.12*	0.11
Interpersonal index trauma	43 (16.5%)	n/a	0.24***	0.27***	0.22***	0.26***
Perceived Social Support	69.7 (12.8)	25–84	−0.09	−0.18*	−0.20**	−0.19*
Cognitive factors						
Post-traumatic dissociation	1.4 (2.3)	0–12	0.46***	0.37***	0.40***	0.41***
Data-driven processing	15.7 (6.1)	7–28	0.48***	0.34***	0.40***	0.43***
Trauma Memory Quality	21.9 (6.8)	11–44	0.58***	0.43***	0.49***	0.52***
Trauma-related appraisals	37.6 (14.3)	25–90	0.58***	0.54***	0.66***	0.65***
Rumination	7.5 (2.8)	3–12	0.57***	0.46***	0.55***	0.58***
Self-blame	3.6 (2.1)	2–8	−0.002	0.30***	0.15*	0.11
Subjective event severity and fear response factors (all peritraumatic)						
Panic	3.6 (2.4)	0–10	0.47***	0.45***	0.45***	0.48***
Perceived life threat	1.9 (1.1)	1–4	0.28***	0.30***	0.35***	0.31***
Perceived harm	2.9 (1.0)	1–4	0.26***	0.24***	0.23**	0.17*
Fear	3.0 (1.1)	1–4	0.43***	0.20**	0.30***	0.35***
Dissociation	4.0 (3.1)	0–12	0.34***	0.30***	0.38***	0.35***
Objective event severity factors						
Pain	3.0 (1.1)	1–4	0.19**	0.19**	0.22**	0.23**
Admission to hospital	73 (28.1%)	n/a	−0.13*	−0.09	−0.11	−0.11
Head injury	97 (38.2%)	n/a	0.02	0.08	0.10	0.08
Number of injuries	1.7 (0.9)	0–5	0.001	0.15*	0.06	0.03
Opiates given in ED	44 (17.9%)	n/a	−0.04	−0.08	−0.08	−0.06

*Note*. *p < 0.05, **p < 0.01, ***p < 0.001.

**Fig. 1.**
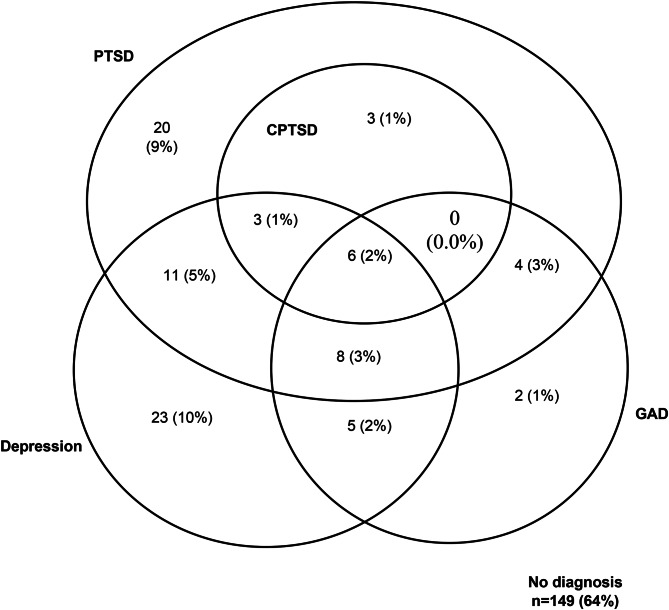
Venn diagram summarizing number of participants meeting criteria for likely diagnoses of PTSD, CPTSD, depression, and GAD at nine weeks post-trauma.

At nine weeks post-trauma, 55 (23.5%) participants met criteria for PTSD, 20 (8.5%) exhibited CPTSD DSO (disturbances of self-organization) symptoms, and 12 (5.2%) met criteria for full CPTSD, according to the measures generated in accordance with ICD-11 criteria (See online Supplementary Table S5). Fifty-six individuals (23.9%) scored highly on the SMFQ to indicate likely depression, and 25 (10.7%) scored highly on the SCAS subscale to indicate likely GAD (see Fig. 1). The requirement for core PTSD symptoms alongside DSO symptoms to meet CPTSD diagnostic criteria meant that, by definition, no participants would fall into the ‘CPTSD only’ category. Eighty-four participants (36%) met the threshold for at least one of the four diagnoses at T2.

### Correlates of psychopathology

The correlation statistics between T1 putative predictor variables and the four T2 outcome variables are displayed in Table 1. Cognitive variables and subjective event severity factors showed the highest correlations with the four psychopathology outcome variables. Trauma memory qualities and trauma-related appraisals were the highest correlated of the predictor factors with PTSD (*r*s = 0.58). Appraisals and rumination were the highest correlated factors with CPTSD (*r*s > 0.46). Depression and GAD scores were also most highly correlated with trauma-related appraisals and rumination (*r*s > 0.55).

### Predictors of PTSD

The psychosocial model was significant and accounted for 5.4% of the variance in PTSD symptom severity (adjusted *R*^2^ = 0.054; see Table 2 for all model fit statistics), with female gender (*β* = 0.14) and interpersonal index trauma (*β* = 0.26) as significant predictors (see online Supplementary Table S6). The objective event severity model was significant and accounted for the smallest variance (adjusted *R*^2^ = 0.03) in PTSD symptom severity of all four models, with pain being the only significant predictor (*β* = 0.21). The subjective event severity model accounted for 33% of variance (adjusted *R*^2^ = 0.33); panic (*β* = 0.37), feeling scared (*β* = 0.15) and dissociation (*β* = 0.23) during the trauma were significant predictors of PTSD. The cognitive model accounted for the greatest variance in PTSD symptom severity (adjusted *R*^2^ = 0.55); greater post-traumatic dissociation (*β* = 0.17), poorer trauma memory quality (*β* = 0.20) and maladaptive appraisals of the trauma (*β* = 0.33) were associated with increased PTSD symptoms. See Table 3 for summary of variables accounting for unique variance in PTSD and the other three outcomes.

**Table 2.** Overall goodness of fit and model statistics for multiple linear regression analyses of predictors of each disorder

Disorder	Model	Adj *R*^2^	*p*	AIC	BIC
**Core PTSD**	Psychosocial	0.054	0.018	1251.87	1284.60
	**Cognitive**	**0.551**	**<0.001**	**1168.24**	**1141.77**
	SES	0.326	<0.001	1227.94	1251.13
	OES	0.030	0.061	1223.17	1245.89
**CPTSD cluster**	Psychosocial	0.132	<0.001	1062.96	1100.69
	**Cognitive**	**0.585**	**<0.001**	**949.54**	**976.01**
	SES	0.291	<0.001	1061.44	1084.63
	OES	0.041	0.026	1046.12	1068.85
**Depression**	Psychosocial	0.128	<0.001	1198.39	1231.07
	**Cognitive**	**0.559**	**<0.001**	**1097.82**	**1124.24**
	SES	0.235	<0.001	1213.16	1236.31
	OES	0.084	<0.008	1171.83	1194.52
**GAD**	Psychosocial	0.125	<0.001	1414.54	1447.21
	**Cognitive**	**0.558**	**<0.001**	**1327.55**	**1353.98**
	SES	0.280	<0.001	1424.85	1448.00
	OES	0.091	<0.001	1382.14	1404.83

SES, Subjective event severity; OES, Objective event severity. Model with fit indices suggesting the best goodness of fit and highest variance in outcome accounted for highlighted in bold. N observations included in each model analysis varied as such: psychosocial *n* = 194; cognitive *n* = 201; SES *n* = 202; OES *n* = 189.

**Table 3.** Variables accounting for unique variance for each outcome

	Variable class			
Outcome	Psychosocial	Cognitive	Subjective event severity	Objective event severity
Core PTSD	Female gender (0.14) Interpers. trauma (0.26)	Dissociation ^a^ (0.17) Memory quality (0.20) Appraisals (0.33) Rumination (0.12)	Panic ^b^ (0.37) Felt scared (0.15) Dissociation ^b^ (0.23)	Pain ^b^ (0.21)
CPTSD cluster	Interpers. trauma (0.31)	Dissociation ^a^ (0.18) Appraisals (0.56) Self-blame (0.27)	Panic ^b^ (0.41) Dissociation (0.20)	Pain ^b^ (0.20)
Depression	Female gender (0.20) Prior traumas (0.16) Interpers. trauma (0.27) Social support (−0.20)	Appraisals (0.68)	Panic ^b^ (0.30) Dissociation ^b^ (0.23)	Pain ^b^ (0.26) Head injury (0.17)
GAD	Female gender (0.24) Interpers. trauma (0.25)	Dissociation ^a^ (0.17) Appraisals (0.51)	Panic ^b^ (0.34) Dissociation ^b^ (0.23)	Pain ^b^ (0.29)

*Note*. ^a^Ongoing; ^b^ Peri-traumatic. Standardized regression (i.e. beta) coefficients are displayed in parentheses.

### Predictors of CPTSD

The psychosocial model was significant and accounted for greater (13%) variance in DSO symptom severity (adjusted *R*^2^ = 0.13) than PTSD (see online Supplementary Table S7). Experiencing interpersonal index trauma (*β* = 0.31) was associated with increased DSO symptoms. Within the subjective event severity model, panic (*β* = 0.41) and dissociation (*β* = 0.20) were significant predictors of later DSO symptoms; this model again accounting for greater variance (adjusted *R*^2^ = 0.29) than the objective model (adjusted *R*^2^ = 0.04), where only pain (*β* = 0.20) accounted for unique variance. A large proportion of variance was accounted for by the cognitive model (adjusted *R*^2^ = 0.59), with post-traumatic dissociation (*β* = 0.18), maladaptive appraisals (*β* = 0.56) and self-blame (*β* = 0.14) acting as significant predictors.

### Predictors of depression

Female gender (*β* = 0.20), prior traumas (*β* = 0.16), interpersonal index trauma (*β* = 0.27), and poorer perceived social support (*β* = −0.20) were significant predictors of later depression in the psychosocial model (adjusted *R*^2^ = 0.13; see online Supplementary Table S8). Within the cognitive model, only increased maladaptive trauma appraisals was a significant predictor of later depression symptoms, with a large coefficient (*β* = 0.68), but the model still accounted for 56% of the variance in depression at week nine (adjusted *R*^2^ = 0.56). Panic (*β* = 0.30) and dissociation during the trauma (*β* = 0.23) were significant predictors within the subjective event severity model (adjusted *R*^2^ = 0.24); pain (*β* = 0.26) and sustaining a head injury (*β* = 0.17) were significant predictors within the objective event severity model (adjusted *R*^2^ = 0.08).

### Predictors of GAD

Female gender (*β* = 0.24) and experiencing an interpersonal index trauma (*β* = 0.25) significantly predicted later GAD symptoms in the psychosocial model (adjusted *R*^2^ = 0.13; see online Supplementary Table S9). The cognitive model showed strong predictive power (adjusted *R*^2^ = 0.56), with trauma appraisals (*β* = 0.51) and dissociation (*β* = 0.17) significant predictors. The subjective event severity model accounted for the second greatest amount of variance in symptoms (adjusted *R*^2^ = 0.28), with panic (*β* = 0.34) and peritraumatic dissociation (*β* = 0.23) as significant predictors. Within the objective event severity model (adjusted *R*^2^ = 0.09), increased pain was a significant predictor of GAD symptom severity (*β* = 0.29).

### Overall model comparisons

Table 2 summarizes the goodness of fit statistics for each model predicting each disorder. Each model was significant. The cognitive model consistently accounted for the greatest variance in symptoms and achieved the best (lowest) AIC and BIC statistics. The subjective event severity model was consistently the second best-fitting model, followed by the psychosocial model. Comparison across disorders indicates that the psychosocial model was comparably stronger in predicting CPTSD, depression, and GAD but weaker in predicting PTSD. The subjective event severity model was stronger in predicting core PTSD and weakest in predicting depression, and the objective model was comparable in predicting depression and GAD but weakest in predicting core PTSD.

### Sensitivity analyses

Further models were evaluated to confirm the robustness of our findings. Given the strong relationship between exposure to interpersonal violence and PTSD (Alisic et al., 2014), the cognitive, subjective event severity, and objective event severity models were re-run, but with interpersonal index event also included in the model. The addition of interpersonal index did not add more than 2% additional variance explained to any of the cognitive models. An additional 5% of variance was accounted for when adding interpersonal violence to the subjective event severity model for CPTSD, but for other outcomes there was only 2–3% variance accounted for. The objective event severity models were improved by 2–8%, with the model for CPTSD the most strongly improved (see online Supplementary Table S10 for revised model fit statistics). Interpersonal index event accounted for unique variance in several models (the objective and subjective event severity models for PTSD, depression and GAD; all revised CPTSD models); moreover, no variables that previously accounted for unique variance lost their significance, with the exception of head injury in the objective event severity model for depression (see online Supplementary Tables S11–S14).

Further models were run to evaluate whether the cognitive model was as robust even when psychosocial processes were included in the same model. Adding psychosocial processes to the cognitive model did not increase the proportion of variance explained by more than 2% for each outcome (see online Supplementary Table S10); moreover, no cognitive variables that previously accounted for unique variance in an outcome ceased to do so (see online Supplementary Tables S15–S18). The same process was repeated but entering the subjective event severity variables alongside the cognitive variables. These models did not account for much additional variance compared to the cognitive variables alone model (see online Supplementary Table S10); the cognitive variables that previously accounted for unique variance continued to do so (see online Supplementary Tables S19–S22).

## Discussion

### Overall findings

In youth exposed to single event trauma, longitudinal models comprising peri- and post-traumatic factors were more powerful predictors of all mental health outcomes than the psychosocial model (primarily pre-trauma factors) at a two month follow up assessment. The cognitive model provided the best model fit for PTSD and CPTSD, and cognitive factors were more powerful predictors than event-related measures. These findings were consistent with our first two hypotheses. However, the cognitive model also derived the best model fit over other models for depression and GAD; this generalized power did not support hypothesis three (that the cognitive model would differentiate between disorders). Overall, poor disorder specificity was indicated, with a similar pattern of goodness of fit indices and some overlap in significant predictors of disorders.

### Understanding CPTSD in children

As a relatively new diagnosis, there are few studies of CPTSD in youth. This study provides some evidence for PTSD and CPTSD being related but distinct presentations in youth, with different predictors and correlates. Trauma memory quality and rumination were not related to CPTSD symptoms in our regression models but were related to PTSD; self-blame accounted for unique variance on CPTSD but not PTSD. Theories of CPTSD have referred to the role of disruption of attachments which leads to the DSO symptoms (Cloitre et al., 2009). This was not supported in our data, however; while social support was modestly negatively correlated with CPTSD symptoms (i.e. a potential protective effect) social support did not play a role in our regression model of CPTSD. Female gender and prior poor well-being were not found to be predictive of CPTSD in our regression model, in contrast to previous research (e.g. Sachser et al., 2017), although this may be due to features of the sample or the well-being measure.

### The predictive power of the cognitive model

The cognitive model of predictors based on Ehlers and Clark's (2000) model of PTSD demonstrated the best model fit indices and greatest proportion of variance accounted for in PTSD, CPTSD, depression, and GAD. Maladaptive appraisals of a traumatic event were a strong cognitive predictor of all disorders, but variation in the significance and strength of other cognitive predictors highlighted some differentiation in the predictive power and applicability of this model to different disorders. For example, all cognitive factors had significant or near to significant roles in predicting greater likelihood of ‘core’ PTSD symptoms, whereas data-driven processing, trauma memory quality, and rumination had no effect in predicting CPTSD symptoms; moreover, self-blame was only related to CPTSD symptoms. This finding supports the validity of ‘core’ and ‘complex’ PTSD as distinct presentations and the use of cognitive behavioral therapy to effectively treat a range of diagnoses experienced by young people (Jensen et al., 2014; Rith-Najarian et al., 2019). Similarly, only greater trauma-related misappraisals were significantly predictive of depression and GAD. Interestingly, rumination appeared to have the most relevance as a predictive factor for core PTSD and showed little predictive value for depression. Rumination is a cognitive process which has been implicated as a core maintaining feature of both GAD and depression (Papageorgiou, 2006); it showed a significant correlation with depressive symptoms but appeared not to predict severity of symptoms nine weeks post-trauma when set against other cognitive factors.

The cognitive model of PTSD may be challenged due to its similar estimates of variance accounted for and model fit indices for all disorders, suggesting poor specificity. However, all disorders studied have theoretical models implicating cognitive factors, with overlap across disorder specific models. The model specificity and goodness of fit results within this research may have reflected greater differences between disorders had we developed different models defining more specific cognitive features of each disorder *a priori*, demonstrated by Ehring et al. (2008). This highlights the importance of assessing if children present with maladaptive cognitive processes given their transdiagnostic significance, and exploring these processes to elucidate to which specific symptoms they may be most vulnerable.

### Psychosocial and event-related predictors of psychopathology following trauma

Experiencing an interpersonal index trauma (rather than an RTA or other accidental injury) appeared to lead to increased risk for all psychopathology. Younger age was not a significant predictor of any disorder, consistent with Trickey et al. (2012). Both CPTSD and depression were predicted by poor perceived social support, highlighting the relevance of good interpersonal networks as a protective factor. Experiencing prior traumas also predicted CPTSD and depression but not GAD or PTSD; multiple childhood traumas have been implicated in developmental research exploring later psychopathology, in a dose-response relationship (e.g. Steine *et al*. 2017; Turgoose, Wilkinson, Shevlin, & Karatzias, 2024). The development of GAD and PTSD may be less related to a disruption in development caused by early traumas.

There was a clear distinction between the relative predictive power of objective *v.* subjective event severity markers, with subjective experiences of greater fear, panic, and perceived threat during the trauma showing greater relevance in predicting later psychopathology than markers of injury severity or requirement for hospital admission. Perceived life threat was not found to be predictive of any disorders, in contrast to previous research (e.g. Cox et al., 2008). Feeling scared, panicked, or dissociating at the time of the event appeared more important, suggesting the emotional experience and fear response is more indicative of later psychopathology than threat appraisals. Peritraumatic pain was a significant predictor of all disorders, although this was measured post-trauma due to limited hospital data, and so could have been a proxy of the child's post-traumatic appraisal of the event.

### Limitations and future directions

While this study is novel, a larger sample may have allowed for the use of structural equation modelling methods to identify outcomes more coherently; it could be that the four outcomes considered here do not accurately represent the range of symptoms that participants experienced. When planning for this study, CPTSD was not yet a diagnostic category, meaning a validated measure of CPTSD was not available. Our study supports a distinction between PTSD and CPTSD within youth, and the importance of assessing and treating maladaptive cognitive processes to potentially reduce the distressing symptoms of CPTSD, PTSD, depression, or anxiety. This field requires further exploration. In particular, we would stress that the nature of the present sample (i.e. children and adolescents recruited from emergency departments) means our findings cannot be generalized to children with multiple trauma exposure (e.g. maltreatment) or other forms of single event trauma (e.g. witnessing an event happening to someone else). Moreover, in seeking to evaluate the role of a wide range of event-related, psychosocial and cognitive process variables, some of the measures used were brief and may not have captured important aspects of the factor being considered (e.g. life stressors in the year prior to the index trauma was based on a frequency of events, and may not have captured the significance or impact of any specific life event).

## Conclusions

These findings present a key addition to understanding the predictors of PTSD and related disorders in youth. The results support the cognitive model of PTSD, but also highlight a lack of disorder specificity of the model. Consideration of disorder-specific cognitions, or the prediction of an overall ‘distress’ factor post-trauma, may be pertinent in further exploration. Overall, the significance of subjective peritraumatic factors and post-traumatic cognitive processes consistently demonstrated the importance of assessing how a child experienced an event in understanding their potential susceptibility to psychopathological symptoms.

## Supporting information

Memarzia et al. supplementary material Memarzia et al. supplementary material

## Supplementary material

The supplementary material for this article can be found at https://doi.org/10.1017/S0033291724001648
