# Supplementary material for: Predictive models of post-traumatic stress disorder, complex post-traumatic stress disorder, depression, and anxiety in children and adolescents following a single-event trauma
Source: Psychol Med. 2024 Oct 7;54(12):3407–16. doi: 10.1017/S0033291724001648 (PMC11496237; doi:10.1017/S0033291724001648)
Supplement: Memarzia et al. supplementary material [file S0033291724001648sup001.docx]

# Supplementary Materials

**Supplementary Table 1:** Exclusion data

| **Reason for Exclusion** | **Number Excluded** |
| --- | --- |
| Intellectual disability | 5 |
| Non-fluent in English | 5 |
| Unconsciousness >15 mins post-event | 0 |
| History of brain damage/brain injury due to trauma | 1 |
| Assault involving caregiver/close relative as the assailant | 0 |
| Ongoing exposure to threat | 0 |
| Significant risk of self-harm/A&E attendance due to self-harm | 1 |
| Under the care of social services/child protection issue | 0 |
| Current symptoms of PTSD related to previous trauma | 0 |
| No parent/guardian consent | 2 |
| Not criterion A trauma | 16 |

**Supplementary Table 2:** Summary of measures or items used for each predictor variable within each model

| **Model & Factors** | **Measure** |
| --- | --- |
| *Psychosocial* | |
| Age  Gender (female)  Mother’s education  Interpersonal index trauma  Prior trauma (lifetime frequency)  Prior life stressors (past year frequency)  Prior well-being concerns  Perceived social support | Sociodemographic questionnaire  Semi-structured interview with parent  Information gathered at admission in ED  Semi-structured interview with parent  Semi-structured interview with parent  Semi-structured interview with parent  MSPSS total score |
| *Cognitive Model* | |
| Post-traumatic dissociation  Data-driven processing  Trauma memory quality  Trauma-related appraisals  Rumination  Self-blame | CPSS post-trauma dissociation items total score  CDDPQ total score  TMQQ total score  CPTCI total score  CRSQ items 1-3 total score  CRSQ items 4-5 total score |
| *Conditioned Fear / Subjective event severity* | |
| Peri-traumatic panic  Peri-traumatic perceived life threat  Peri-traumatic perceived harm  Peri-traumatic fear  Peri-traumatic dissociation | CPP total score  CPT: item 1 ‘thought I will die’  CPT: item 2 ‘thought I would be badly hurt’  CPT: item 3 ‘very scared’  CPSS peritraumatic dissociation items total score |
| *Objective event severity* | |
| Pain  Number of injuries sustained  Head injury sustained  Admitted to hospital  Opiates administered in ED | Child Pain Scale (peritraumatic)  Information recorded by nurses during admission to ED |

**Supplementary Table 3:** Prior trauma parent interview

| Over the course of [insert child’s name] life, has he/she experienced any of the following events? |
| --- |
| Questions |
| a. Serious accident, fire, or explosion (for example, an industrial, farm, car, plane, or boating accident) |
| b. Natural disaster (for example, tornado, hurricane, flood, or major earthquake) |
| c. Non-sexual assault by a family member or someone the child knew (for example, being mugged, physically attacked, shot, stabbed, or held at gunpoint) |
| d. Non-sexual assault by a stranger (for example, being mugged, physically attacked, shot, stabbed, or held at gunpoint) |
| e. Unwanted sexual assault by a family member or someone the child knew (for example, rape or attempted rape) |
| f. Unwanted sexual assault by a stranger (for example, rape or attempted rape) |
| g. Military combat or a war zone |
| h. Sexual contact with someone who was 5 or more years older than your child (for example, contact with genitals, breasts) |
| i. Imprisonment (for example, prison inmate, prisoner of war, hostage) |
| k. Torture |
| k. Life-threatening illness |
| l. Death of a family member or friend |
| m. Other traumatic event (please specify) |

Note. Taken from the Posttraumatic Diagnostic Scale (PDS-5, Foa, Cashman, Jaycox, and Perry, 1997; Foa et al., 2016).

**Supplementary Table 4:** Items used to generate a measure of ICD-11 PTSD and CPTSD

| ICD-11 symptom | Item selected |
| --- | --- |
| **PTSD** | |
| *Re-experiencing* | |
| Flashbacks | CPSS item 3: Acting or feeling as if the event was happening again (hearing something or seeing a picture about it and feeling as if I am there again) |
| Intrusive memories | CPSS item 1: Having upsetting thoughts or images about the event that came into your head when you didn’t want them to |
| Nightmares | CPSS item 2: Having bad dreams or nightmares |
| Fear, horror, physical sensations or same emotions as during event | CPSS item 4: Feeling upset when you think or hear about the event (for example, feeling scared, angry, sad, guilty etc).  CPSS item 5: Having feelings in your body when you think about or hear about the event (for example, breaking out in a sweat, heart beating fast). |
| *Avoidance* | |
| Of thoughts or memories | CPSS item 6: Trying not to think about, talk about, or have feelings about the event. |
| Of activities, situations or people | CPSS item 7: Trying to avoid activities, people, or places that remind you of the traumatic event. |
| *Current threat perception* | |
| Hypervigilance | CPSS item 16: Being overly careful (for example, checking to see who is around you and what is around you). |
| Enhanced startle response | CPSS item 17: Being jumpy or easily startled (for example, when someone walks up behind you). |
| **Complex PTSD** | |
| *Affect regulation problems* | |
| Anger | CPSS item 14: Feeling irritable or having fits of anger. |
| Violent or reckless behaviour | CPSS item 21: Taking more risks and being reckless or dangerous. |
| Emotional reactivity, or a lack of emotion | CPSS item 11: Not being able to have strong feelings (for example, being unable to cry or unable to feel very happy). |
| *Negative beliefs about self* | |
| Diminished, or defeated | CPSS item 12: Feeling as if your future plans or hopes will not come true (for example, you will not have a job or get married or have kids). |
| Worthless | CPTCI item 7: I am no good |
| Feelings of shame, guilt, or failure (related to the event) | CRSQ 5: It was my fault the event happened |
| *Interpersonal difficulties* | |
| Difficulties sustaining relationships | CPTCI item 5: I don’t trust other people |
| Difficulties feeling close to others | CPSS item 10: Not feeling close to people around you. |

Note. All items were scored 0-3.

**Supplementary Table 5:** Frequency of participants meeting symptom and diagnostic criteria at week two and week nine post-trauma

| Symptom/diagnosis | T1 (Week 2)  n=217 | T2 (Week 9)  n=234 |
| --- | --- | --- |
| **ICD-11 PTSD** | | |
| *Re-experiencing* |  | |
| Flashbacks (CPSS 3) | 76 35% | 62 26.5% |
| Intrusive memories (CPSS 1) | 117 53.9% | 87 37.2% |
| Nightmares (CPSS 2) | 75 34.6% | 62 26.5% |
| Fear, horror, physical sensations (CPSS 4)  or same emotions as during event (CPSS 5) | 118 54.4%  71 32.7% | 83 35.5%  49 21.9% |
| *Re-experiencing criteria met*  *(1 or 2 or 3, and 4 or 5)* | *108 49.8%* | *76 32.5%* |
| *Avoidance* |  |  |
| Of thoughts or memories (CPSS 6) | 108 49.8% | 79 33.7% |
| Of activities, situations or people (CPSS 7) | 70 32.3% | 59 25.2% |
| *Avoidance criteria met (6 or 7)* | *119 54.8%* | *94 40.2%* |
| *Current threat perception* |  |  |
| Hypervigilance (CPSS 16) | 119 54.8% | 94 40.2% |
| Enhanced startle response (CPSS 17) | 82 37.8% | 68 29.1% |
| *Threat criteria met (16 or 17)* | *131 61.5%* | *110 47%* |
| ***ICD-11 PTSD criteria met***  Score of 1 or higher on items (1 or 2 or 3) +  (4 or 5) + (6 or 7) + (16 or 17) | **73 33.6%** | **55 23.5%** |
| **ICD-11 Complex PTSD** | | |
| *Affect regulation problems* |  |  |
| Anger (CPSS 14) | 80 36.9% | 57 24.4% |
| Violent or reckless behaviour (CPSS 21) | 26 11.9% | 26 11.1% |
| Emotional reactivity or lack of emotion (CPSS 11) | 46 21.2% | 34 14.5% |
| *Affect regulation criteria met (14 or 21 or 11)* | *101 46.5%* | *80 34.2%* |
| *Negative beliefs about self* |  |  |
| Diminished or defeated (CPSS 12) | 30 13.8% | 23 9.8% |
| Worthlessness (CPTCI 7) | 41 18.9% | 52 22.2% |
| Guilt, shame or failure (CRSQ 5) | 90 41.5% | 82 35% |
| *Negative beliefs criteria met (12 or 7, and 5)* | *37 17.1%* | *34 14.5%* |
| *Interpersonal difficulties* |  |  |
| Difficulties sustaining relationships (CPTCI 5) | 80 36.9% | 92 39.3% |
| Difficulties feeling close to others (CPSS 10) | 45 20.7% | 37 15.8% |
| *Interpersonal difficulties criteria met* | 91 41.9% | 99 42.3% |
| ***ICD-11 CPTSD symptom cluster criteria met*** | **25 11.5%** | **20 8.5%** |
| **Full ICD-11 CPTSD diagnostic criteria met**  *(core PTSD symptoms plus CPTSD cluster)* | **18 8.3%** | **12 5.1%** |
| **Depression**  *(SMFQ total score cut-off 8/<)* | **54 24.9%** | **56 24%** |
| **Generalised Anxiety Disorder**  *(SCAS GAD t-score cut-off)* | **29 13.4%** | **25 10.7%** |

####

#### **Supplementary Table 6:** Linear regression model statistics for predictors of PTSD

| **Model** | **Predictor** | **Unstandardised Coefficient** | **Standardised coefficient** | **p** | **lower 95%CI** | **upper 95%CI** |
| --- | --- | --- | --- | --- | --- | --- |
| PSYCHOSOCIAL | Age | -.102 | -.050 | .497 | -.388 | .167 |
|  | **Female gender** | **1.713** | **.142** | **.055** | **.001** | **3.403** |
|  | Mother's education | .745 | .061 | .394 | -.892 | 2.450 |
|  | Prior traumas | .287 | .048 | .514 | -.522 | 1.065 |
|  | Prior life stressors | .229 | .042 | .580 | -.519 | .996 |
|  | **Interpersonal index trauma** | **4.242** | **.259** | **.001** | **2.072** | **6.736** |
|  | Prior wellbeing concerns | .687 | .048 | .519 | -1.302 | 2.625 |
|  | Perceived social support | -.015 | -.033 | .654 | -.085 | .052 |
| COGNITIVE | **Dissociation (ongoing)** | **.425** | **.167** | **.006** | **.114** | **.706** |
|  | Data-driven processing | .106 | .108 | .061 | -.004 | .212 |
|  | **Trauma memory quality** | **.175** | **.201** | **.004** | **.060** | **.281** |
|  | **Trauma appraisals** | **.136** | **.331** | **.000** | **.078** | **.196** |
|  | **Rumination** | **.257** | **.122** | **.069** | **.005** | **.526** |
|  | Self-blame | -.246 | -.083 | .086 | -.519 | .024 |
| SUBJECTIVE EVENT SEVERITY | **Peri-traumatic panic** | **.917** | **.368** | **<.001** | **.554** | **1.267** |
|  | Perceived life threat | .183 | .033 | .651 | -.617 | .954 |
|  | Perceived harm | -.336 | -.057 | .436 | -1.169 | .485 |
|  | **Felt scared** | **.842** | **.150** | **.051** | **.017** | **1.661** |
|  | **Peri-traumatic dissociation** | **.439** | **.227** | **.001** | **.214** | **.680** |
| OBJECTIVE EVENT SEVERITY | **Peri-traumatic pain** | **1.173** | **.212** | **.004** | **.345** | **1.895** |
|  | Admitted to hospital | -1.333 | -.099 | .206 | -3.290 | .698 |
|  | Head injury | .817 | .066 | .387 | -.928 | 2.672 |
|  | Number of injuries sustained | -.066 | -.010 | .898 | -1.048 | .947 |
|  | Given opiates in ED | -.143 | -.009 | .905 | -2.392 | 2.263 |

Note. Variables highlighted in bold are where the bootstrapped 95% confidence does not cross the line of no effect.

#### **Supplementary Table 7:** Linear regression model statistics for predictors of Complex PTSD

| **Model** | **Predictor** | **Unstandardised coefficient** | **Standardised coefficient** | **p** | **lower 95%CI** | **upper 95%CI** |
| --- | --- | --- | --- | --- | --- | --- |
| PSYCHOSOCIAL | Age | .054 | .040 | .566 | -.128 | .223 |
|  | Female gender | .894 | .114 | .108 | -.144 | 1.938 |
|  | Mother's education | .155 | .020 | .776 | -.820 | 1.198 |
|  | Prior traumas | .482 | .125 | .080 | -.048 | 1.011 |
|  | Prior life stressors | -.063 | -.018 | .806 | -.553 | .434 |
|  | **Interpersonal index trauma** | **3.327** | **.312** | **<.001** | **1.892** | **4.886** |
|  | Prior wellbeing concerns | .839 | .091 | .208 | -.352 | 2.206 |
|  | Perceived social support | -.037 | -.125 | .082 | -.076 | .003 |
| COGNITIVE | **Dissociation (ongoing)** | **.290** | **.176** | **.003** | **.106** | **.478** |
|  | Data-driven processing | -.013 | -.020 | .714 | -.076 | .060 |
|  | Trauma memory quality | .008 | .015 | .821 | -.065 | .080 |
|  | **Trauma appraisals** | **.150** | **.563** | **<.001** | **.115** | **.188** |
|  | Rumination | .123 | .091 | .158 | -.045 | .288 |
|  | **Self-blame** | **.265** | **.139** | **.003** | **.092** | **.440** |
| SUBJECTIVE EVENT SEVERITY | **Peri-traumatic panic** | **.663** | **.412** | **<.001** | **.424** | **.888** |
|  | Perceived life threat | .173 | .049 | .519 | -.360 | .687 |
|  | Perceived harm | .123 | .032 | .666 | -.449 | .692 |
|  | Felt scared | -.096 | -.026 | .737 | -.607 | .493 |
|  | **Peri-traumatic dissociation** | **.253** | **.202** | **.002** | **.091** | **.414** |
| OBJECTIVE EVENT SEVERITY | **Peri-traumatic pain** | **.700** | **.200** | **.006** | **.177** | **1.192** |
|  | Admitted to hospital | -.949 | -.112 | .152 | -2.190 | .337 |
|  | Head injury | .864 | .110 | .146 | -.199 | 1.984 |
|  | Number of injuries sustained | .118 | .027 | .715 | -.528 | .719 |
|  | Given opiates in ED | -.444 | -.046 | .556 | -1.800 | 1.003 |

Note. Variables highlighted in bold are where the bootstrapped 95% confidence does not cross the line of no effect.

#### **Supplementary Table 8:** Linear regression model statistics for predictors of Depression

| **Model** | **Predictor** | **Unstandardised Coefficient** | **Standardised coefficient** | **p** | **lower 95%CI** | **upper 95%CI** |
| --- | --- | --- | --- | --- | --- | --- |
| PSYCHOSOCIAL FACTORS  AND STRESSORS | Age | .016 | .008 | .906 | -.245 | .272 |
|  | **Female gender** | **2.268** | **.204** | **.004** | **.880** | **3.781** |
|  | Mother's education | .557 | .049 | .474 | -.851 | 2.037 |
|  | **Prior traumas** | **.862** | **.158** | **.028** | **.144** | **1.638** |
|  | Prior life stressors | -.123 | -.025 | .736 | -.747 | .550 |
|  | **Interpersonal index trauma** | **4.111** | **.269** | **<.001** | **2.019** | **6.335** |
|  | Prior wellbeing concerns | .171 | .013 | .857 | -1.639 | 2.000 |
|  | **Perceived social support** | **-.086** | **-.203** | **.005** | **-.142** | **-.027** |
| COGNITIVE | Dissociation (ongoing) | .088 | .038 | .532 | -.168 | .353 |
|  | Data-driven processing | .039 | .043 | .452 | -.053 | .135 |
|  | Trauma memory quality | -.032 | -.040 | .563 | -.136 | .068 |
|  | **Trauma appraisals** | **.258** | **.683** | **<.001** | **.207** | **.310** |
|  | Rumination | .135 | .070 | .292 | -.104 | .393 |
|  | Self-blame | .140 | .052 | .282 | -.094 | .374 |
| SUBJECTIVE EVENT SEVERITY | **Peri-traumatic panic** | **.686** | **.301** | **<.001** | **.372** | **1.026** |
|  | Perceived life threat | .624 | .124 | .117 | -.108 | 1.436 |
|  | Perceived harm | -.159 | -.030 | .706 | -.951 | .651 |
|  | Felt scared | .021 | .004 | .959 | -.734 | .833 |
|  | **Peri-traumatic dissociation** | **.402** | **.227** | **.001** | **.152** | **.652** |
| OBJECTIVE EVENT SEVERITY | **Peri-traumatic pain** | **1.328** | **.260** | **<.001** | **.593** | **2.012** |
|  | Admitted to hospital | -1.620 | -.132 | .084 | -3.324 | .105 |
|  | **Head injury** | **1.926** | **.170** | **.023** | **.316** | **3.535** |
|  | Number of injuries sustained | -.410 | -.065 | .377 | -1.209 | .503 |
|  | Given opiates in ED | -.940 | -.067 | .378 | -2.863 | 1.207 |

Note. Variables highlighted in bold are where the bootstrapped 95% confidence does not cross the line of no effect.

#### **Supplementary Table 9:** Linear regression model statistics for predictors of GAD

| **Model** | **Predictor** | **Unstandardised Coefficient** | **Standardised coefficient** | **p** | **lower 95%CI** | **upper 95%CI** |
| --- | --- | --- | --- | --- | --- | --- |
| PSYCHOSOCIAL | Age | .213 | .064 | .361 | -.217 | .661 |
|  | **Female gender** | **4.662** | **.241** | **.001** | **2.101** | **7.294** |
|  | Mother's education | 2.103 | .107 | .122 | -.560 | 4.659 |
|  | Prior traumas | .995 | .104 | .145 | -.340 | 2.352 |
|  | Prior life stressors | .665 | .076 | .298 | -.511 | 2.026 |
|  | **Interpersonal index trauma** | **6.576** | **.247** | **.001** | **2.853** | **1.379** |
|  | Prior wellbeing concerns | .685 | .030 | .679 | -2.405 | 3.964 |
|  | Perceived social support | -.057 | -.077 | .282 | -.159 | .046 |
| COGNITIVE | **Dissociation (ongoing)** | **.704** | **.173** | **.005** | **.199** | **1.180** |
|  | Data-driven processing | .084 | .053 | .359 | -.076 | .250 |
|  | Trauma memory quality | .031 | .022 | .749 | -.157 | .225 |
|  | **Trauma appraisals** | **.338** | **.514** | **<.001** | **.248** | **.429** |
|  | Rumination | .381 | .113 | .093 | -.035 | .816 |
|  | Self-blame | -.194 | -.041 | .399 | -.650 | .292 |
| SUBJECTIVE EVENT SEVERITY | **Peri-traumatic panic** | **1.342** | **.338** | **<.001** | **.767** | **1.903** |
|  | Perceived life threat | 1.124 | .128 | .094 | -.209 | 2.511 |
|  | Perceived harm | -.681 | -.073 | .340 | -2.124 | .653 |
|  | Felt scared | .432 | .048 | .543 | -1.019 | 1.845 |
|  | **Peri-traumatic dissociation** | **.701** | **.227** | **.001** | **.274** | **1.142** |
| OBJECTIVE EVENT SEVERITY | **Peri-traumatic pain** | **2.565** | **.287** | **<.001** | **1.369** | **3.777** |
|  | Admitted to hospital | -2.726 | -.127 | .096 | -5.929 | .253 |
|  | Head injury | 2.785 | .140 | .058 | -.039 | 5.577 |
|  | Number of injuries sustained | -1.334 | -.121 | .100 | -2.867 | .328 |
|  | Given opiates in ED | -1.408 | -.058 | .449 | -4.897 | 2.218 |

Note. Variables highlighted in bold are where the bootstrapped 95% confidence does not cross the line of no effect.

**Supplementary Table 10:** Overall goodness of fit and model statistics for multiple linear regression analyses of predictors of each disorder: sensitivity analyses.

| **Disorder** | **Model** | **Adj R^2^** | ***p*** | **AIC** | **BIC** |
| --- | --- | --- | --- | --- | --- |
| **Core PTSD** | Psychosocial | .054 | 0.018 | 1251.87 | 1284.60 |
|  | Psychosocial + cognitive | .555 | <0.001 | 1100.76 | 1152.96 |
|  | Cognitive ^a^ | .550 | <0.001 | 1143.29 | 1173.07 |
|  | SES ^a^ | .359 | <0.001 | 1218.89 | 1245.40 |
|  | SES + cognitive | .556 | <0.001 | 1144.02 | 1187.03 |
|  | OES ^a^ | .071 | .003 | 1215.92 | 1421.90 |
| **CPTSD cluster** | Psychosocial | .132 | <0.001 | 1062.96 | 1100.69 |
|  | Psychosocial + cognitive | .607 | <0.001 | 910.77 | 962.97 |
|  | Cognitive ^a^ | .597 | <0.001 | 944.16 | 973.93 |
|  | SES ^a^ | .347 | <0.001 | 1045.52 | 1072.03 |
|  | SES + cognitive | .588 | <0.001 | 952.80 | 995.81 |
|  | OES ^a^ | .121 | <0.001 | 1030.60 | 1056.58 |
| **Depression** | Psychosocial | .128 | <0.001 | 1198.39 | 1231.07 |
|  | Psychosocial + cognitive | .570 | <0.001 | 1056.78 | 1108.90 |
|  | Cognitive ^a^ | .557 | <0.001 | 1099.42 | 1129.15 |
|  | SES ^a^ | .261 | <0.001 | 1207.04 | 1233.51 |
|  | SES + cognitive | .552 | <0.001 | 1105.64 | 1148.58 |
|  | OES ^a^ | .104 | <0.001 | 1168.61 | 1194.54 |
| **GAD** | Psychosocial | .125 | <0.001 | 1414.54 | 1447.21 |
|  | Psychosocial + cognitive | .569 | <0.001 | 1271.11 | 1323.23 |
|  | Cognitive ^a^ | .543 | <0.001 | 1328.74 | 1358.47 |
|  | SES ^a^ | .314 | <0.001 | 1416.03 | 1442.49 |
|  | SES + cognitive | .538 | <0.001 | 1334.89 | 1377.84 |
|  | OES ^a^ | .130 | <0.001 | 1400.79 | 1374.86 |

*SES=Subjective event severity; OES=Objective event severity. Model with fit indices suggesting the best goodness of fit and highest variance in outcome accounted for highlighted in bold.* ^a^ *Adjusting for interpersonal trauma (the psychosocial always included interpersonal trauma vs other).*

#### **Supplementary Table 11:** Linear regression model statistics for predictors of PTSD, with the addition of interpersonal trauma

| Model | Predictor | Unstandardised Coefficient | Standardised coefficient | p | lower 95%CI | upper 95%CI |
| --- | --- | --- | --- | --- | --- | --- |
| COGNITIVE | **Dissociation (ongoing)** | **.419** | **.165** | **.007** | **.127** | **.729** |
|  | Data-driven processing | **.109** | **.110** | **.056** | **.001** | **.209** |
|  | **Trauma memory quality** | **.176** | **.203** | **.004** | **.056** | **.297** |
|  | **Trauma appraisals** | **.132** | **.320** | **<.001** | **.073** | **.193** |
|  | Rumination | .256 | .122 | .070 | -.008 | .517 |
|  | Self-blame | -.230 | -.078 | .113 | -.518 | .052 |
|  | Interpersonal index trauma | .539 | .034 | .499 | -.886 | 2.008 |
| SUBJECTIVE EVENT SEVERITY | **Peri-traumatic panic** | **.831** | **.334** | **<.001** | **.479** | **1.154** |
|  | Perceived life threat | .217 | .039 | .582 | -.483 | .981 |
|  | Perceived harm | -.361 | -.062 | .391 | -1.194 | .457 |
|  | **Felt scared** | **1.174** | **.209** | **.007** | **.363** | **2.013** |
|  | **Peri-traumatic dissociation** | **.374** | **.193** | **.003** | **.134** | **.629** |
|  | **Interpersonal index trauma** | **3.119** | **.196** | **.001** | **1.215** | **5.040** |
| OBJECTIVE EVENT  SEVERITY | Peri-traumatic pain | **1.184** | **.214** | **.003** | **.441** | **1.948** |
|  | Admitted to hospital | -.707 | -.053 | .501 | -2.476 | 1.272 |
|  | Head injury | -.246 | -.020 | .804 | -2.178 | 1.640 |
|  | Number of injuries sustained | -.038 | -.006 | .940 | -1.025 | 1.002 |
|  | Given opiates in ED | .005 | .000 | .996 | -1.982 | 2.256 |
|  | Interpersonal index trauma | **3.705** | **.234** | **.003** | **1.335** | **6.102** |

Note. Variables where the 95% CI of the bootstrapped regression coefficient did not cross zero are highlighted in bold.

#### **Supplementary Table 12:** Linear regression model statistics for predictors of CPTSD, with the addition of interpersonal trauma

| Model | Predictor | Unstandardised Coefficient | Standardised coefficient | p | lower 95%CI | upper 95%CI |
| --- | --- | --- | --- | --- | --- | --- |
| COGNITIVE | **Dissociation (ongoing)** | **.275** | **.167** | **.004** | **.101** | **.458** |
|  | Data-driven processing | -.008 | -.012 | .827 | -.072 | .055 |
|  | Trauma memory quality | .013 | .023 | .731 | -.060 | .084 |
|  | **Trauma appraisals** | **.139** | **.522** | **<.001** | **.105** | **.178** |
|  | Rumination | .122 | .090 | .156 | -.032 | .282 |
|  | **Self-blame** | **.304** | **.159** | **.001** | **.148** | **.482** |
|  | **Interpersonal index trauma** | **1.306** | **.127** | **.008** | **.399** | **2.254** |
| SUBJECTIVE EVENT SEVERITY | **Peri-traumatic panic** | **.591** | **.367** | **<.001** | **.371** | **.835** |
|  | Perceived life threat | .201 | .057 | .434 | -.303 | .693 |
|  | Perceived harm | .102 | .027 | .709 | -.417 | .606 |
|  | Felt scared | .182 | .050 | .517 | -.378 | .733 |
|  | **Peri-traumatic dissociation** | **.198** | **.158** | **.014** | **.043** | **.361** |
|  | **Interpersonal index trauma** | **2.613** | **.254** | **<.001** | **1.492** | **3.773** |
| OBJECTIVE EVENT  SEVERITY | **Peri-traumatic pain** | **.709** | **.203** | **.004** | **.241** | **1.128** |
|  | Admitted to hospital | -.414 | -.049 | .521 | -1.600 | .873 |
|  | Head injury | -.045 | -.006 | .942 | -1.212 | 1.143 |
|  | Number of injuries sustained | .141 | .033 | .648 | -.418 | .740 |
|  | Given opiates in ED | -.317 | -.033 | .661 | -1.558 | 1.053 |
|  | **Interpersonal index trauma** | **3.166** | **.317** | **<.001** | **1.807** | **4.782** |

Note. Variables where the 95% CI of the bootstrapped regression coefficient did not cross zero are highlighted in bold.

#### **Supplementary Table 13:** Linear regression model statistics for predictors of depression, with the addition of interpersonal trauma

| Model | Predictor | Unstandardised Coefficient | Standardised coefficient | p | lower 95%CI | upper 95%CI |
| --- | --- | --- | --- | --- | --- | --- |
| COGNITIVE | Dissociation (ongoing) | .084 | .036 | .552 | -.191 | .386 |
|  | Data-driven processing | .041 | .045 | .431 | -.056 | .139 |
|  | Trauma memory quality | -.031 | -.039 | .577 | -.137 | .070 |
|  | **Trauma appraisals** | **.255** | **.674** | **<.001** | **.202** | **.312** |
|  | Rumination | .135 | .070 | .293 | -.115 | .390 |
|  | Self-blame | .152 | .056 | .248 | -.099 | .386 |
|  | Interpersonal index trauma | .448 | .030 | .540 | -1.100 | 1.828 |
| SUBJECTIVE EVENT SEVERITY | **Peri-traumatic panic** | **.618** | **.271** | **.001** | **.264** | **.945** |
|  | Perceived life threat | .638 | .126 | .103 | -.048 | 1.372 |
|  | Perceived harm | -.177 | -.033 | .670 | -1.008 | .626 |
|  | Felt scared | .304 | .059 | .475 | -.504 | 1.213 |
|  | **Peri-traumatic dissociation** | **.347** | **.196** | **.005** | **.111** | **.572** |
|  | **Interpersonal index trauma** | **2.655** | **.180** | **.005** | **.843** | **4.648** |
| OBJECTIVE EVENT  SEVERITY | **Peri-traumatic pain** | **1.320** | **.259** | **<.001** | **.632** | **2.007** |
|  | Admitted to hospital | -1.206 | -.098 | .202 | -2.916 | .573 |
|  | Head injury | 1.211 | .107 | .174 | -.544 | 2.805 |
|  | Number of injuries sustained | -.367 | -.058 | .424 | -1.273 | .599 |
|  | Given opiates in ED | -.843 | -.060 | .425 | -2.765 | 1.150 |
|  | **Interpersonal index trauma** | **2.507** | **.172** | **.025** | **.469** | **4.589** |

Note. Variables where the 95% CI of the bootstrapped regression coefficient did not cross zero are highlighted in bold.

#### **Supplementary Table 14:** Linear regression model statistics for predictors of GAD, with the addition of interpersonal trauma

| Model | Predictor | Unstandardised Coefficient | Standardised coefficient | p | lower 95%CI | upper 95%CI |
| --- | --- | --- | --- | --- | --- | --- |
| COGNITIVE | **Dissociation (ongoing)** | **.694** | **.170** | **.006** | **.253** | **1.207** |
|  | Data-driven processing | .088 | .056 | .333 | -.091 | .272 |
|  | Trauma memory quality | .034 | .024 | .728 | -.161 | .212 |
|  | **Trauma appraisals** | **.329** | **.500** | **<.001** | **.232** | **.424** |
|  | Rumination | .381 | .113 | .094 | -.023 | .850 |
|  | Self-blame | -.163 | -.034 | .485 | -.596 | .313 |
|  | Interpersonal index trauma | 1.137 | .044 | .380 | -1.188 | 3.773 |
| SUBJECTIVE EVENT SEVERITY | **Peri-traumatic panic** | **1.209** | **.305** | **<.001** | **.600** | **1.786** |
|  | Perceived life threat | 1.151 | .131 | .079 | -.088 | 2.506 |
|  | Perceived harm | -.715 | -.076 | .305 | -2.030 | .538 |
|  | Felt scared | .981 | .109 | .170 | -.409 | 2.332 |
|  | **Peri-traumatic dissociation** | **.593** | **.192** | **.004** | **.196** | **.984** |
|  | **Interpersonal index trauma** | **5.162** | **.201** | **.001** | **2.253** | **8.386** |
| OBJECTIVE EVENT  SEVERITY | **Peri-traumatic pain** | **2.546** | **.285** | **<.001** | **1.297** | **3.676** |
|  | Admitted to hospital | -1.769 | -.082 | .277 | -4.818 | 1.108 |
|  | Head injury | 1.132 | .057 | .460 | -1.822 | 3.958 |
|  | Number of injuries sustained | -1.234 | -.112 | .120 | -2.642 | .382 |
|  | Given opiates in ED | -1.181 | -.048 | .517 | -4.405 | 2.466 |
|  | **Interpersonal index trauma** | **5.798** | **.226** | **.003** | **2.301** | **9.569** |

Note. Variables where the 95% CI of the bootstrapped regression coefficient did not cross zero are highlighted in bold.

#### **Supplementary Table 15:** Linear regression model statistics for cognitive and psychosocial predictors of PTSD (single model)

| Predictor class | Predictor | Unstandardised Coefficient | Standardised coefficient | p | lower 95%CI | upper 95%CI |
| --- | --- | --- | --- | --- | --- | --- |
| PSYCHOSOCIAL | Age | -.097 | -.047 | .358 | -.297 | .087 |
|  | Female gender | -.378 | -.031 | .558 | -1.576 | .804 |
|  | Mother's education | .675 | .055 | .273 | -.550 | 1.754 |
|  | Prior traumas | -.310 | -.052 | .316 | -.868 | .275 |
|  | Prior life stressors | -.126 | -.023 | .678 | -.680 | .474 |
|  | Interpersonal index trauma | .444 | .027 | .632 | -1.285 | 2.129 |
|  | Prior wellbeing concerns | .399 | .028 | .597 | -.922 | 1.771 |
|  | Perceived social support | .018 | .039 | .459 | -.030 | .063 |
| COGNITIVE | **Dissociation (ongoing)** | **.599** | **.231** | **<.001** | **.273** | **.952** |
|  | Data-driven processing | .094 | .094 | .115 | -.018 | .198 |
|  | **Trauma memory quality** | **.174** | **.199** | **.006** | **.064** | **.288** |
|  | **Trauma appraisals** | **.135** | **.330** | **<.001** | **.078** | **.194** |
|  | Rumination | .218 | .102 | .137 | -.064 | .495 |
|  | Self-blame | -.279 | -.095 | .066 | -.577 | .003 |

Note. All variables were entered simultaneously. Variables where the 95% CI of the bootstrapped regression coefficient did not cross zero are highlighted in bold.

#### **Supplementary Table 16:** Linear regression model statistics for cognitive and psychosocial predictors of CPTSD (single model)

| Predictor class | Predictor | Unstandardised Coefficient | Standardised coefficient | p | lower 95%CI | upper 95%CI |
| --- | --- | --- | --- | --- | --- | --- |
| PSYCHOSOCIAL | Age | .054 | .040 | .403 | -.066 | .181 |
|  | Female gender | -.380 | -.048 | .337 | -1.183 | .383 |
|  | Mother's education | -.235 | -.030 | .532 | -.944 | .484 |
|  | Prior traumas | .180 | .047 | .341 | -.197 | .537 |
|  | Prior life stressors | -.172 | -.049 | .356 | -.528 | .169 |
|  | **Interpersonal index trauma** | **1.112** | **.105** | **.051** | **.067** | **2.098** |
|  | Prior wellbeing concerns | .284 | .031 | .537 | -.534 | 1.133 |
|  | Perceived social support | -.001 | -.004 | .934 | -.029 | .027 |
| COGNITIVE | **Dissociation (ongoing)** | **.375** | **.223** | **<.001** | **.192** | **.594** |
|  | Data-driven processing | -.023 | -.035 | .530 | -.091 | .041 |
|  | Trauma memory quality | .015 | .027 | .687 | -.059 | .086 |
|  | **Trauma appraisals** | **.137** | **.512** | **<.001** | **.098** | **.172** |
|  | Rumination | .109 | .078 | .223 | -.051 | .258 |
|  | **Self-blame** | **.271** | **.141** | **.004** | **.113** | **.440** |

Note. All variables were entered simultaneously. Variables where the 95% CI of the bootstrapped regression coefficient did not cross zero are highlighted in bold.

#### **Supplementary Table 17:** Linear regression model statistics for cognitive and psychosocial predictors of depression (single model)

| Predictor class | Predictor | Unstandardised Coefficient | Standardised coefficient | p | lower  95%CI | upper  95%CI |
| --- | --- | --- | --- | --- | --- | --- |
| PSYCHOSOCIAL | Age | -.004 | -.002 | .964 | -.181 | .176 |
|  | Female gender | .564 | .051 | .335 | -.557 | 1.592 |
|  | Mother's education | .174 | .015 | .755 | -.904 | 1.180 |
|  | Prior traumas | .452 | .083 | .108 | -.058 | 1.006 |
|  | Prior life stressors | -.108 | -.022 | .697 | -.606 | .418 |
|  | Interpersonal index trauma | .770 | .051 | .364 | -.864 | 2.434 |
|  | Prior wellbeing concerns | -.862 | -.066 | .211 | -2.050 | .389 |
|  | **Perceived social support** | **-.051** | **-.121** | **.021** | **-.094** | **-.011** |
| COGNITIVE | Dissociation (ongoing) | .103 | .043 | .512 | -.216 | .419 |
|  | Data-driven processing | .059 | .064 | .272 | -.052 | .165 |
|  | Trauma memory quality | -.049 | -.061 | .388 | -.160 | .056 |
|  | **Trauma appraisals** | **.244** | **.646** | **<.001** | **.187** | **.304** |
|  | Rumination | .159 | .081 | .231 | -.096 | .408 |
|  | Self-blame | .089 | .033 | .515 | -.152 | .357 |

Note. All variables were entered simultaneously. Variables where the 95% CI of the bootstrapped regression coefficient did not cross zero are highlighted in bold.

#### **Supplementary Table 18:** Linear regression model statistics for cognitive and psychosocial predictors of GAD (single model)

| Predictor class | Predictor | Unstandardised Coefficient | Standardised coefficient | p | lower  95% CI | upper  95% CI |
| --- | --- | --- | --- | --- | --- | --- |
| PSYCHOSOCIAL | Age | 0.205 | 0.061 | 0.220 | -0.120 | 0.526 |
|  | Female gender | 1.655 | 0.085 | 0.106 | -0.380 | 3.639 |
|  | Mother's education | 1.733 | 0.088 | 0.077 | -0.054 | 3.530 |
|  | Prior traumas | 0.204 | 0.021 | 0.677 | -0.674 | 1.091 |
|  | Prior life stressors | 0.239 | 0.027 | 0.622 | -0.577 | 1.113 |
|  | Interpersonal index trauma | 0.759 | 0.029 | 0.608 | -1.890 | 3.678 |
|  | Prior wellbeing concerns | -0.259 | -0.011 | 0.829 | -2.398 | 2.030 |
|  | Perceived social support | 0.003 | 0.005 | 0.929 | -0.073 | 0.078 |
| COGNITIVE | Dissociation (ongoing) | **0.803** | **0.192** | **0.004** | **0.286** | **1.305** |
|  | Data-driven processing | 0.100 | 0.062 | 0.289 | -0.081 | 0.278 |
|  | Trauma memory quality | 0.011 | 0.008 | 0.914 | -0.183 | 0.199 |
|  | Trauma appraisals | **0.314** | **0.476** | **0.000** | **0.223** | **0.407** |
|  | Rumination | **0.427** | **0.125** | **0.065** | **0.027** | **0.894** |
|  | Self-blame | -0.215 | -0.045 | 0.370 | -0.643 | 0.285 |

Note. All variables were entered simultaneously. Variables where the 95% CI of the bootstrapped regression coefficient did not cross zero are highlighted in bold.

#### **Supplementary Table 19:** Linear regression model statistics for cognitive and subjective event severity predictors of PTSD (single model)

| Predictor class | Predictor | Unstandardised Coefficient | Standardised coefficient | p | lower  95% CI | upper  95% CI |
| --- | --- | --- | --- | --- | --- | --- |
| COGNITIVE | **Dissociation (ongoing)** | **.399** | **.157** | **.014** | **.107** | **.703** |
|  | Data-driven processing | .089 | .090 | .191 | -.033 | .212 |
|  | **Trauma memory quality** | **.166** | **.192** | **.006** | **.050** | **.283** |
|  | **Trauma appraisals** | **.130** | **.315** | **<.001** | **.072** | **.188** |
|  | Rumination | .286 | .136 | .054 | .010 | .546 |
|  | **Self-blame** | **-.309** | **-.104** | **.037** | **-.609** | **-.031** |
| SES | **Peri-traumatic panic** | **.335** | **.135** | **.041** | **.041** | **.624** |
|  | **Perceived life threat** | **-.685** | **-.124** | **.048** | **-1.359** | **-.037** |
|  | Perceived harm | .021 | .004 | .953 | -.651 | .746 |
|  | Felt scared | .215 | .038 | .557 | -.481 | .928 |
|  | Peri-traumatic dissociation | -.035 | -.018 | .776 | -.256 | .195 |

Note. All variables were entered simultaneously. Variables where the 95% CI of the bootstrapped regression coefficient did not cross zero are highlighted in bold. SES = Subjective event severity.

#### **Supplementary Table 20:** Linear regression model statistics for cognitive and subjective event severity predictors of CPTSD (single model)

| Predictor class | Predictor | Unstandardised Coefficient | Standardised coefficient | p | lower  95% CI | upper  95% CI |
| --- | --- | --- | --- | --- | --- | --- |
| COGNITIVE | **Dissociation (ongoing)** | **.255** | **.155** | **.012** | **.052** | **.436** |
|  | Data-driven processing | -.014 | -.022 | .744 | -.095 | .066 |
|  | Trauma memory quality | .010 | .019 | .782 | -.054 | .085 |
|  | **Trauma appraisals** | **.146** | **.549** | **.000** | **.111** | **.183** |
|  | Rumination | .129 | .095 | .161 | -.048 | .304 |
|  | **Self-blame** | **.224** | **.117** | **.015** | **.064** | **.387** |
| SES | **Peri-traumatic panic** | **.216** | **.135** | **.034** | **.010** | **.416** |
|  | Perceived life threat | -.216 | -.061 | .315 | -.616 | .169 |
|  | Perceived harm | .150 | .039 | .508 | -.276 | .548 |
|  | Felt scared | -.296 | -.081 | .194 | -.712 | .134 |
|  | Peri-traumatic dissociation | -.009 | -.007 | .905 | -.155 | .138 |

Note. All variables were entered simultaneously. Variables where the 95% CI of the bootstrapped regression coefficient did not cross zero are highlighted in bold. SES = Subjective event severity.

#### **Supplementary Table 21:** Linear regression model statistics for cognitive and subjective event severity predictors of depression (single model)

| Predictor class | Predictor | Unstandardised Coefficient | Standardised coefficient | p | lower  95% CI | upper  95% CI |
| --- | --- | --- | --- | --- | --- | --- |
| COGNITIVE | Dissociation (ongoing) | .063 | .027 | .673 | -.224 | .344 |
|  | Data-driven processing | .053 | .059 | .396 | -.064 | .178 |
|  | Trauma memory quality | -.026 | -.033 | .639 | -.131 | .077 |
|  | **Trauma appraisals** | **.256** | **.677** | **<.001** | **.203** | **.312** |
|  | Rumination | .156 | .081 | .253 | -.099 | .435 |
|  | Self-blame | .139 | .051 | .309 | -.114 | .414 |
| SES | Peri-traumatic panic | .099 | .043 | .511 | -.179 | .388 |
|  | Perceived life threat | .012 | .002 | .970 | -.556 | .627 |
|  | Perceived harm | -.056 | -.010 | .868 | -.708 | .574 |
|  | Felt scared | -.393 | -.076 | .243 | -1.022 | .238 |
|  | Peri-traumatic dissociation | .005 | .003 | .966 | -.214 | .227 |

Note. All variables were entered simultaneously. Variables where the 95% CI of the bootstrapped regression coefficient did not cross zero are highlighted in bold. SES = Subjective event severity.

#### **Supplementary Table 22:** Linear regression model statistics for cognitive and subjective event severity predictors of GAD (single model)

| Predictor class | Predictor | Unstandardised Coefficient | Standardised coefficient | p | lower  95% CI | upper  95% CI |
| --- | --- | --- | --- | --- | --- | --- |
| COGNITIVE | **Dissociation (ongoing)** | **.656** | **.161** | **.014** | **.205** | **1.196** |
|  | Data-driven processing | .099 | .063 | .373 | -.104 | .299 |
|  | Trauma memory quality | .028 | .020 | .776 | -.145 | .223 |
|  | **Trauma appraisals** | **.330** | **.502** | **<.001** | **.237** | **.421** |
|  | Rumination | .419 | .125 | .085 | -.005 | .898 |
|  | Self-blame | -.216 | -.046 | .371 | -.658 | .224 |
| SES | Peri-traumatic panic | .357 | .090 | .181 | -.137 | .869 |
|  | Perceived life threat | -.187 | -.021 | .741 | -1.234 | .805 |
|  | Perceived harm | -.254 | -.027 | .668 | -1.348 | .793 |
|  | Felt scared | -.356 | -.040 | .550 | -1.439 | .746 |
|  | Peri-traumatic dissociation | -.056 | -.018 | .782 | -.440 | .302 |

Note. All variables were entered simultaneously. Variables where the 95% CI of the bootstrapped regression coefficient did not cross zero are highlighted in bold. SES = Subjective event severity.
